# Next-Generation Genotyping: Innovations Driving Plant Genomic Improvement

**DOI:** 10.3390/life16030521

**Published:** 2026-03-21

**Authors:** Valeriya Kostyukova, Roza Kenzhebekova, Egor Protsenko, Bakyt Dulat, Marina Khusnitdinova, Dilyara Gritsenko

**Affiliations:** 1 Laboratory of Molecular Biology, Institute of Plant Biology and Biotechnology, Almaty 050040, Kazakhstanrozakenzhebekova344@gmail.com (R.K.); germironame@gmail.com (M.K.); 2 Research Center AgriBioTech, Almaty 050040, Kazakhstan; 3 Faculty of Biology and Biotechnology, Al-Farabi Kazakh National University, Almaty 050040, Kazakhstan

**Keywords:** GBS, genomic selection (GS), genotyping, graph genomes, RAD-seq, SNP arrays, whole-genome sequencing (WGS)

## Abstract

In recent years, plant genotyping has been shifting from the accumulation of whole-genome data toward their effective use in breeding programs This review examines key genotyping platforms, including single-nucleotide polymorphism (SNP) arrays, reduced-representation sequencing methods such as genotyping-by-sequencing (GBS) and restriction site-associated DNA sequencing (RAD-seq), targeted genotyping approaches, and whole-genome sequencing (WGS), analyzing their informativeness, cost, and computational limitations. The transition to pangenome-based genotyping and graph genomes is discussed, as these approaches reduce reference bias and increase sensitivity for detecting structural variants, introgressions, and rare alleles that are important for adaptation and breeding. The growing role of AI/ML is highlighted in modeling complex genotype–phenotype relationships, integrating genomic and phenotypic data, and improving the accuracy and interpretability of genomic predictions.

## 1. Introduction

Plant genotyping is one of the key tools of modern plant genomics and breeding. It enables the identification of polymorphisms associated with productivity, disease resistance, and adaptation to stresses, as well as the establishment of causal relationships between DNA variation and phenotype. Such an approach significantly accelerates the selection of agriculturally valuable forms. In the context of climate change and the need to enhance food security, rapid, accurate, and scalable assessment of genetic diversity is becoming an especially important task [1]. Advances in sequencing methods and the reduction in their cost have made it possible to move from the analysis of individual loci to the study of variation at the whole-genome level. This, in turn, has opened new opportunities for identifying genes controlling quantitative traits and complex adaptive mechanisms.

Traditional molecular methods such as RFLP, AFLP, and SSR played a key role in the early stages of plant genetics; however, they have a number of limitations. The main drawback of these approaches is limited genome coverage. In addition, RFLP and AFLP methods can be technically demanding and often demonstrate lower reproducibility compared with modern genotyping approaches [2]. Moreover, traditional approaches are poorly suited to polyploid or large genomes, where sequence homology interferes with accurate allele identification [3]. These limitations make traditional methods insufficiently informative for modern breeding tasks, which has driven the transition to next-generation sequencing (NGS) technologies.

High-density SNP arrays, genotyping-by-sequencing, and whole-genome resequencing have enabled comprehensive detection of genetic variants across the genome in wheat [4] and maize [5], substantially increasing marker density and the accuracy of association mapping. Bread wheat (*Triticum aestivum*), a hexaploid with a large and repeat-rich genome, serves as an excellent model for studying polyploid genomics and complex trait architecture. The transition to SNP-based genotyping has made it possible to identify more than 260 million polymorphisms in modern cultivars, landraces, and wild relatives, providing a level of resolution in genomic diversity that is unattainable with classical markers [6]. In soybean [7] and rapeseed [8], these platforms have facilitated the identification of loci underlying yield components and stress resistance, whereas in vegetatively propagated and polyploid crops such as potato [9], they have improved the resolution of genetic diversity analyses and increased the accuracy of genomic prediction.

The transition to NGS-based methods has become an important stage in the development of genomics. Modern high-throughput sequencing technologies, such as Illumina, PacBio, and Oxford Nanopore Technologies (ONT), make it possible to obtain millions of single-nucleotide polymorphisms (SNPs) and structural variants in a single experiment. Methods such as GBS, RAD-seq, ddRAD-seq, amplicon sequencing, targeted sequencing, and whole-genome sequencing (including low-coverage approaches) make it possible to obtain a sufficiently comprehensive and representative picture of the genome at moderate cost [10]. One of the key advances in modern genotyping has been the introduction and gradual transition to genomic selection (GS). The essence of this approach lies in the use of genome-wide markers to predict the phenotypic potential of plants [11]. It is particularly effective for quantitative traits controlled by many genes with small individual effects, for example, yield, resistance to abiotic stresses, and fruit quality [12,13,14]. GS models make it possible to simultaneously account for the effects of thousands of SNPs and structural variants, thereby increasing selection accuracy and reducing the duration of the breeding cycle [15]. In combination with NGS technologies, this opens up opportunities for faster selection of optimal genotypes in large populations and for high-throughput screening.

The importance of implementing next-generation genotyping is also confirmed by large international projects aimed at studying the genetic diversity of crops. For example, in the 3000 Rice Genomes Project [16], more than three thousand rice accessions were sequenced, which made it possible to identify approximately 19 million SNPs and to determine genes associated with yield and stress resistance. Similar results have been obtained for *Arabidopsis thaliana* [17], where genomic analysis helped reconstruct evolutionary relationships and identify loci involved in adaptation. These examples clearly demonstrate that next-generation genotyping not only deepens the understanding of the genetic basis of traits but also becomes a practical tool for crop improvement.

An important aspect of applying these technologies is their adaptation to different types of plant genome architecture. In this regard, the review examines the methodological principles of NGS-based approaches and their applicability to complex genomes, including polyploid, large, and repeat-rich genomes that pose significant challenges for analysis. Particular attention is given to the role of modern genotyping methods in assessing genetic diversity, conducting genome-wide association studies (GWAS), and implementing genomic selection strategies, which in recent years have become key tools for accelerating the breeding process.

## 2. SNP Arrays as a Tool for High-Throughput Plant Genotyping and Genomic Selection

With the introduction of high-throughput plant genotyping, approaches to studying genomes have changed, substantially accelerating the application of genomics in breeding. As a result, there has been a need for reliable and standardized methods that provide reproducible, highly accurate data on polymorphisms across the genomes of different crops. One of the most widely used solutions has been platforms based on the detection of previously known variants. SNP arrays (SNP chips) are high-throughput genotyping platforms based on DNA hybridization to preselected oligonucleotide probes specific to particular SNP positions [18]. The ability to detect tens of thousands to millions of SNPs across the genome in parallel has driven the widespread use of this method [19] for constructing genetic maps [20], GWAS [21], and population structure analysis [22]. Since the first SNP chips were introduced, marker panels have expanded considerably, covering thousands of new loci. As a result, high-density panels have been developed for major crops: 660 K markers for hexaploid wheat [20], 618 K for soybean [23], 480 K for apple [24], 700 K for rice [25], and 600 K for maize [26]. These panels provide high resolution for studying population genetic structure and conducting genomic selection.

A number of specialized platforms are used to read SNP-array data and perform large-scale plant genotyping. Platforms based on DNA microarray technologies and sequencing are most widely used for genotyping, as they provide high reproducibility and throughput. For the analysis of polymorphisms in plant genomes, microarray scanning systems (e.g., iScan, NextScan, HiScan) as well as automated stations for microarray hybridization and signal detection (e.g., Axiom arrays using the GeneTitan station) have been successfully applied [27,28,29,30,31]. As an alternative for lower-density SNP genotyping, PCR-based systems are used, such as the QuantStudio 12 K Flex Real-Time PCR System from Thermo Fisher Scientific (USA) [32]. Although the density of such panels is lower than that of conventional chips, these approaches provide flexibility in studies where the focus needs to be placed on a limited set of functional or QTL-linked markers.

The widespread implementation of SNP arrays has become a foundation for the transition from traditional breeding to integrated genomic strategies. The use of these technologies makes it possible to develop crop-wide panels, accelerate genomic selection, improve the accuracy of estimating the genetic value of lines, control genetic purity, and track introgressions from wild relatives. In addition, SNP chips have become the basis for building GWAS resources and QTL maps, which substantially expand the possibilities for analyzing functional genome variation [33]. A major limitation of the method is ascertainment bias, associated with the fact that SNPs are selected from a limited panel of founder genomes, which reduces analytical accuracy for other genetic groups or closely related species [34]. Because chips contain predefined polymorphisms, they capture only the variants included during array design, limiting the discovery of new or rare alleles in populations with high genetic diversity [35]. Moreover, high-density chips are costly and are intended for processing large numbers of samples, making them economically unjustified for small-scale studies.

Currently, we observe that large standard panels are no longer available in the open catalogs of the main companies that work with SNP arrays. At present, predominantly custom designs are offered. This observation raises several questions: how widely commercial SNP panels are used, whether there is a shift toward user-defined solutions, and which genotyping platform is most in demand today. In addition, it remains unclear whether the reduction in open catalogs reflects the discontinuation of large fixed arrays or a transition to more flexible, sequencing-oriented genotyping methods. Table 1 presents the number of original research publications for the major agricultural crops. An extended version of the table with the full list of sources is provided in Supplementary File S1.

The absolute leader is the 90 K iSelect panel (141 publications), followed by MaizeSNP50 (123 publications) and BARCSoySNP6k (58 publications). Publication activity does not show a direct dependence on marker density. The most widely used panels are not necessarily the highest-density ones, but rather those that were standardized early and supported by major research consortia (e.g., 90 K iSelect, MaizeSNP50, BARCSoySNP6k). This confirms that the level of technology adoption is determined not only by technical characteristics, but also by the degree of integration into the scientific community.

Table 2 presents the number of custom SNP designs for major crops and the most common marker densities used in the panels. Supplementary File S2 provides a table of references.

An analysis of publications devoted to custom SNP panels demonstrates a pronounced predominance of low- and medium-density panels. The largest number of studies is associated with panels containing 1–12 thousand markers (94 publications) and 13–45 thousand SNPs (83 publications), whereas ultra-high-density panels (>95 thousand) appear in only 14 studies. This pattern of publication activity indicates that in practical breeding and applied genetics, moderate-density panels are predominantly used, as they provide an optimal balance between genotyping cost and informativeness. Unlike large consortium-based arrays (e.g., 90 K iSelect or MaizeSNP50), custom panels are more often developed for specific research objectives and do not always achieve broad international dissemination.

The dynamics of publication activity over time show a gradual increase in the number of studies starting in the late 2000s, peaking around 2018–2019, followed by fluctuations. The growth in publications during 2012–2019 reflects the active implementation of GWAS and genomic selection in breeding programs. The subsequent decline or instability may be associated with a gradual shift in some studies toward sequencing-based approaches (GBS, WGS), which provide a more flexible and scalable framework for variant discoveries.

However, it is important to understand that the transition to next-generation sequencing methods has not displaced SNP arrays; on the contrary, it has substantially simplified and accelerated their development. Massively parallel sequencing has enabled the discovery of millions of SNPs across a wide range of genotypes. As a result, SNP arrays have become more informative, more representative, and better tailored to specific crops and breeding objectives. The logic of these technologies lies in their complementarity: NGS is used at the stage of variant discovery and construction of reference panels, whereas SNP arrays provide highly reproducible, standardized, and cost-effective genotyping of large populations. This is particularly important for genomic selection, assessment of line purity, and long-term monitoring programs.

## 3. Reduced-Representation and Targeted Sequencing in Plant Genotyping

There has been significant interest in SNP markers, and the development of next-generation sequencing techniques has driven the rise of new high-throughput SNP-typing methods. The principle of RAD-seq is that before sequencing, the genome is digested by a single restriction enzyme at specific sites, producing a stable set of fragments for sequencing [36]. RAD-seq provides more predictable genome coverage, but requires more careful library preparation and often a higher per-sample cost [37]. RAD-seq involves digestion of the genome at specific sites using a single restriction enzyme. After restriction, DNA is additionally fragmented (shearing) by mechanical methods, yielding fragments of different lengths around restriction sites. This is followed by size selection, generating a controlled library [36]. The presence of shearing and size-selection steps fundamentally distinguishes RAD-seq from other approaches [38]. A key limitation of RAD-seq applicability to large plant genomes is the loss of loci under selective pressure. Despite relatively even coverage around restriction sites, RAD-seq is more suitable for sequencing relatively simple genomes—with fewer repeats and a more uniform distribution of restriction sites—or for cases where fragment length control is important [39]. Standardized bioinformatic processing can also lead to the loss of important polymorphisms [40].

It should be noted that the initial RAD-seq protocols served as a starting point for the development of a number of derivative methods aimed at increasing data reproducibility, simplifying library preparation, and reducing the cost of analysis. In particular, the 2b-RAD approach was developed, based on the use of type IIB restriction enzymes, which excise short fragments of fixed length around restriction sites [41], allowing the formation of highly standardized libraries and reducing variability in fragment length between samples. The method was successfully tested on an F2 rice population (*O. sativa* L.), where an average of 2,000,332 high-quality reads per sample was obtained with high coverage uniformity [42]. In total, 3598 markers containing 3804 SNPs were identified, with a missing data rate of 18.9%.

Another example is ezRAD, which uses standard library preparation protocols applied on most high-throughput sequencing platforms [43]. The use of universal library preparation kits makes it possible to avoid specialized adapters and complex laboratory procedures characteristic of classical RAD-seq protocols. The ezRAD method was specifically designed for work with organisms that lack complete genomic resources. 

Several modifications and derivatives of RAD-based approaches have been developed to improve reproducibility, scalability, and applicability across different plant systems; key variants are briefly outlined below. In addition, other modifications of RAD approaches have also become widespread. For example, double-digest RAD-seq (ddRAD) uses a combination of two restriction enzymes [44], which allows more precise control over the number of fragments obtained and increases the reproducibility of libraries between experiments [45]. The ddRAD method is characterized by high flexibility and adaptability to various objects and tasks. Jordon-Thaden et al. (2020) showed that the basic protocol with two restriction enzymes works effectively even with herbarium specimens and silica-gel-dried tissues in representatives of four plant genera [46].

Alongside RAD-derived methods, alternative reduced-representation sequencing approaches have been developed, among which genotyping-by-sequencing (GBS) represents a conceptually related but distinct strategy. The emergence and development of alternative genome complexity-reduction methods have been driven by the aim to improve the reproducibility and accuracy of genotyping. Genotyping-by-sequencing (GBS) is one such widely used method. The major benefit of GBS is the simultaneous use of reduced genome representation, NGS, and multiplexing that allows for its broad application to very large and complex genomes in practice at an affordable cost [47]. The approach relies on initial genome complexity reduction by one or more restriction enzymes [48] and subsequent sequencing of the generated fragments employing NGS platforms [49].

Since the first protocol published by Elshire et al. [49], GBS has become one of the most widely applied approaches for studying genetic diversity, mapping quantitative traits, and implementing genomic selection in crop plants [47,50]. One example of large-scale application of GBS is maize (*Z. mays*) research, where this method was used to genotype national and international collections of inbred lines comprising 2815 samples [51]. In that study, GBS was applied to analyze the genetic diversity of the U.S. collection, resulting in the identification of 681,257 SNP markers. These data subsequently formed the basis for GWASs and genomic selection programs, confirming the suitability of GBS for working with large and genetically diverse panels [52,53].

The application of GBS in wheat (*T. aestivum*) is also distinctive when considering its hexaploid nature. Under the CIMMYT program, GBS has been employed for genotyping > 130,000 lines of bread wheat, resulting in 24,069 high-quality SNP markers distributed evenly across the 22 chromosomes [54]. A study of wheat and Barley (*H. vulgare*) genomes was among the initial demonstrations of the GBS approach in plant genomics. The genetic map was generated based on these data, and 34,000 SNPs for barley and over 30,000 SNPs for wheat were mapped; de novo maps without a reference genome were demonstrated [55]. Likewise, GBS has been utilized in a set of crop species such as sorghum [56], maize [57], and legumes [58,59], allowing the identification of polymorphisms associated with agriculturally important traits.

However, GBS is readily affected by errors caused by low read coverage and limited ability to detect true homozygotes [38], often resulting in a large number of missing data points and complicating the identification of true heterozygous genotypes [60]. Because of these challenges, proper genotype imputation and error correction during bioinformatic processing and downstream analyses are important [60]. Moreover, the efficiency of GBS largely depends on the quality of the reference genome [61]. The large genome size (16 Gb) of hexaploid wheat, which contains more than 85% duplicated sequences [62], also increases the likelihood of genotyping errors, because the genome includes a large number of paralogous and homeologous sequences [63]. Despite limitations associated with genome size, GBS remains one of the most cost-effective and scalable approaches for breeding programs.

Targeted next-generation sequencing occupies an intermediate position between whole-genome sequencing and reduced-representation methods and has become an important tool in applied plant genomics. Unlike GBS and RAD-seq, which target random subsets of the genome, targeted approaches focus from the outset on predefined DNA regions of greatest interest for breeding and functional genomics. This can substantially increase genotyping accuracy, simplify data interpretation, and make the method more applicable in practical breeding programs. Among targeted approaches, a fundamental distinction is made between amplicon-oriented methods, based on PCR amplification of preselected loci, and capture-based sequencing methods, which use sets of synthetic oligonucleotide probes for the selective enrichment of target genomic fragments. These strategies differ substantially both in their technical implementation and in the types of tasks for which they are optimal.

Amplicon panels are typically used for high-throughput genotyping of previously known SNP markers in breeding populations, where the analysis of a large number of samples at minimal cost is required [64,65]. Such studies show that amplicon-based approaches are particularly effective for monitoring previously identified functional variants associated with agronomically important traits.

In contrast, hybridization capture methods, such as Hyb-Seq, make it possible not only to genotype known markers but also to identify new polymorphisms within selected genomic regions, which makes them especially useful for studies of genetically diverse populations, phylogenomics, and analyses of gene structural variation [66,67,68]. An analysis of the literature shows that capture-based methods are actively used for sequencing hundreds and thousands of nuclear genes across a wide range of plants, including both model and wild species, and allow the recovery of comparable sets of orthologous sequences even at considerable phylogenetic distances between taxa [69,70,71].

Amplicon-based sequencing is a method in which predefined genomic regions are amplified by PCR, and the resulting amplicons are sequenced [72]. The method can use multiplex panels, enabling simultaneous analysis of dozens to hundreds of loci [73,74]. This approach differs from WGS in its focus: it requires less sequencing, reduces background higher depth, specifically in the regions of interest [75]. In plant breeding and genetic studies, this approach is used to target QTL regions [2,76], genes involved in resistance to biotic and abiotic stresses [64,75,77], and functionally important SNP and InDel markers [78]. This focus enables direct work with loci that have a proven association with traits of agronomic value, which is especially important for marker-assisted selection and validation of candidate genes. A key advantage of amplicon-based approaches is high genotyping accuracy, achieved through deep coverage of each target region and a minimal number of missing data points [64,79]. Unlike GBS, where low coverage can lead to errors in distinguishing homozygous and heterozygous genotypes, amplicon sequencing provides stable and reproducible detection of allelic variants even in complex populations [78]. This makes the method particularly valuable for routine analysis of breeding lines and cultivars.

Amplicon-based genotyping has proven to be a highly accurate and practically oriented approach in real crop improvement programs. For example, Yang et al. demonstrated in grapevine that GBS markers can be converted into multiplex amplicon panels for marker-assisted selection: with coverage depths on the order of hundreds of reads per locus, low missingness and high genotype reproducibility were achieved, enabling effective tracking of QTLs associated with resistance and quality traits even in highly heterozygous populations [64]. The development of this approach, in the form of rhAmpSeq, was demonstrated in another study [65], in which a panel of ~2000 amplicons designed to conserved “core genome” regions demonstrated high marker transferability across Vitis species and cost efficiency at high multiplexing. In soybean (*G. max*), the efficiency of highly multiplexed amplicon sequencing was demonstrated for targeted analysis of genes associated with phenological traits. High sequencing depth ensured low missingness and high genotype reproducibility [80]. In wheat, multiplex genotyping of markers linked to agronomically important traits has been widely applied using high-throughput sequencing-based approaches [81]. Amplicon-based approaches have also been used to identify induced mutations in predefined genes in the flax genome [82]. In crop improvement practice, amplicon sequencing should be viewed as an optimal tool for targeted and reproducible monitoring of known functionally important loci, but not as a universal replacement for broader genomic approaches.

The Hyb-Seq method combines hybridization capture of target regions with high-throughput sequencing and enables the simultaneous analysis of hundreds to thousands of genes [66]. The method includes designing probes (oligonucleotides) complementary to predefined genomic regions (e.g., genes of interest, conserved regions, or QTL-associated loci) [71]. DNA is then fragmented, and probe-bound DNA is identified and isolated. The resulting target fragments are subjected to massively parallel sequencing (Illumina) [83]. The method is applicable in phylogenomics, evolutionary and population studies, and plant breeding, where it is important to examine many genes across species and cultivars [66,71,84]. Another simplified approach is genome skimming. Genome skimming is an independent, low-depth sequencing method; however, within the Hyb-Seq framework, it is used as an additional data source, enabling the simultaneous acquisition of organellar genomes and ribosomal DNA sequences alongside targeted capture of nuclear genes [66]. It involves shallow sequencing of the genome to obtain information on high-copy sequences (chloroplast and mitochondrial genomes, ribosomal repeats) [85]. The method includes DNA library preparation, shotgun sequencing, and bioinformatic analysis. Shotgun sequencing breaks the genome into many random fragments that are sequenced in parallel. This method does not require prior knowledge of genome structure. Using bioinformatic algorithms, reads are aligned and assembled into complete sequences [86]. Given the high copy number of target genes (thousands of copies per cell), a coverage depth of 1–5× is sufficient [87,88,89].

In research devoted to genotyping and the analysis of genetic diversity, terminological confusion between different sequencing approaches is often observed. In particular, GBS and RAD-seq methods, which share a common conceptual basis (restriction-based genome reduction), are frequently used as interchangeable terms, despite substantial differences in protocols, library types, and data reproducibility [90]. Such blurring of terminology complicates comparisons between studies, assessment of method reproducibility, and correct interpretation of the resulting genomic data.

**Figure 1:**
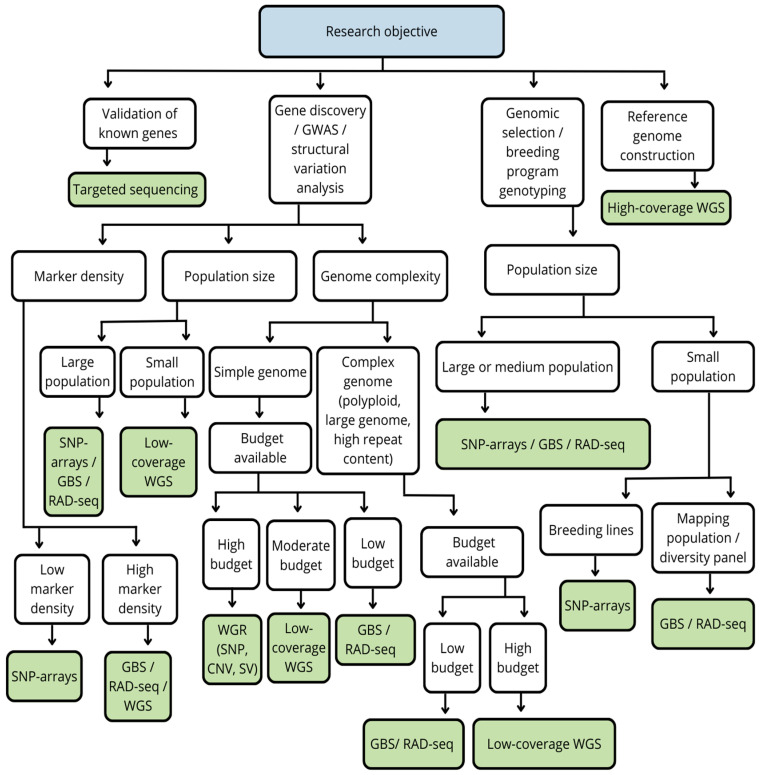
Practical decision tree for selecting appropriate genotyping platforms in plant genomics and breeding. This scheme integrates key experimental factors.

Taken together, the approaches discussed reflect the evolution of plant genotyping methods from large-scale screening of genomic variation to more precise and applied analytical strategies. The rational choice between GBS, RAD-seq, and targeted sequencing is determined by the balance between study scale, genome complexity, and specific breeding objectives, while their combined use allows genomic data to be integrated most effectively into modern crop improvement programs. In Figure 1, we present practical decision pathways for selecting appropriate genotyping technologies depending on the type of research objective, genome complexity, population characteristics, and available resources.

## 4. Whole-Genome Genotyping Strategies in Plant Breeding

Reduced-representation genome methods were developed as a cheaper alternative to whole genome sequencing (WGS) that enable the genotyping of hundreds of samples at orders of magnitude lower costs for sequencing and data analysis.

Whole-genome sequencing, owing to its complete genome coverage, enables analysis of various types of genetic variation, including SNPs [91,92], indels (InDels) [93], structural variants [94], and copy number variation (CNV) [95,96]. WGS can also be used for genomic mapping [97], evolutionary and population genomics [98], as well as functional genomics focusing on genes involved in stress resistance, productivity and other traits [91,99,100]. The benefits of WGS are the ability to capture the whole genome without certainty bias [101]. However, such an approach is severely restricted. Aside from the high cost, whole-genome sequencing generates a large set of data that needs a large amount of computational resources for storage and analysis [102]. In addition, accurate detection of low- and rare-variant heterozygotes is challenging and requires high sequencing depth, thereby increasing costs [103].

Whole-genome resequencing (WGR) is one of the most informative and versatile approaches to genotyping, allowing the analysis of genetic variability with maximum accuracy [98]. In applied genomics, WGR plays a key role, as it serves as the basis for the development of high-density marker panels, including SNP arrays and reference panels for subsequent genotype imputation [18,20,104]. WGR data are used to select informative and evenly distributed markers, which reduces systematic biases and ensures the comparability of genotypic data across different populations and breeding materials [10,77]. At the same time, despite its clear advantages, the widespread use of WGR in breeding programs is still limited by the high cost of sequencing, substantial computational resource requirements, and the complexity of storing and processing large volumes of data, especially for species with large and polyploid genomes.

In response to the limitations of classical WGR, a strong trend has emerged in recent years toward using low-coverage whole-genome sequencing (low-coverage WGS). In this approach, each sample is sequenced at a depth of approximately 0.5–5×, which substantially reduces analysis costs while retaining the ability to obtain genome-wide data [105]. Missing genotype information is reconstructed using high-quality reference panels generated from high-coverage sequencing reads [106]. This approach can be particularly effective for genomic selection tasks, where the key objective is not precise determination of every locus but accurate reconstruction of haplotype structure for phenotype prediction [107]. Low-coverage WGS with imputation demonstrates high predictive ability for quantitative traits [108] and can be considered a compromise between the marker density of WGR and the cost efficiency of reduced-representation methods. In addition, low-coverage WGR can be used to refine genotype identification and sample origin [109].

The development of long-read technologies, represented by the PacBio HiFi and Oxford Nanopore platforms, has opened a new stage in plant genotyping associated with systematic analysis of structural variation. Long-read sequencing enables direct detection of large-scale genome rearrangements, including long deletions and duplications, inversions, complex rearrangements, and copy number variation, which often remain inaccessible to standard NGS approaches [110,111,112,113]. These technologies are particularly important for haplotype genotyping, as read length allows reconstruction of contiguous genomic sequences without the need for complex statistical models [114]. They also facilitate analysis of epigenetic modifications (e.g., methylated bases) without bisulfite conversion. ONT can “read” methylation directly from native sequencing signals [115,116].

**Figure 2:**
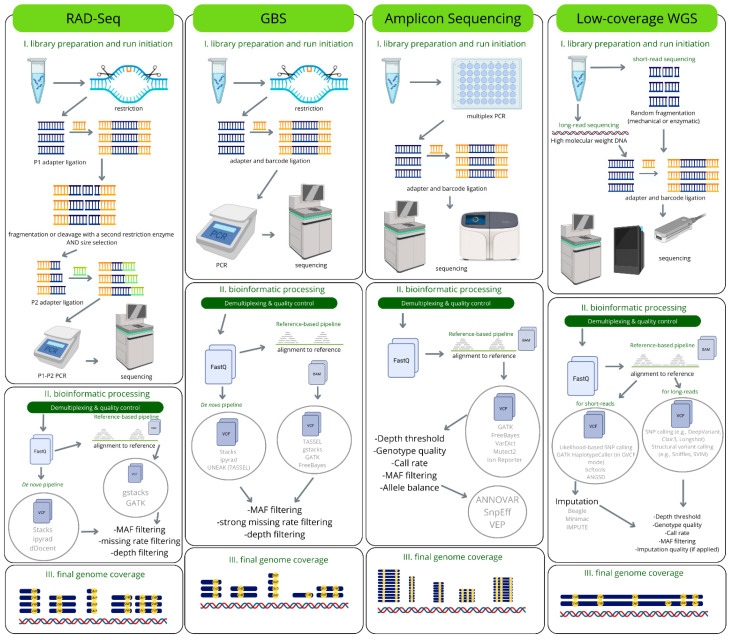
Differences in library preparation and analysis among the methods used in genomic selection.

PacBio is a technology that generates long reads (typically ~10–25 kb) with very high accuracy (~99.8%) [117]. It produces so-called HiFi reads through repeated sequencing of the same molecule (circular consensus sequencing, CCS). The method is based on preparing an SMRTbell library, followed by multiple passes over the same molecule to increase accuracy [118]. ONT is a platform capable of reading very long DNA fragments (sometimes hundreds of kilobases or more), i.e., ultra-long reads [119]. Limitations of these methods include increased requirements for the quality and quantity of input DNA: it must be high-molecular-weight and minimally damaged [85,90]. In addition, the need for substantial computational resources (storage, assembly, long-read alignment, handling large fast5/BAM files) makes these technologies expensive [120]. A brief comparison of the approaches discussed is presented in Figure 2.

## 5. Innovations in Plant Genotyping

In recent years, plant genotyping has been undergoing changes associated with a rethinking of the role of whole-genome sequencing. Although WGS has become the gold standard for detecting all types of genetic variation, its use in large-scale breeding programs remains limited in practice due to high cost and substantial computational burden. As a result, the focus is shifting from simply accumulating whole-genome data to the efficient use of genetic diversity, including structural variants, introgressions, and rare alleles.

The transition to pangenome-based genotyping reflects a fundamental shift in plant genomics—from the use of a single reference genome to representing species-wide genetic diversity as a pangenome that includes both “core” genes shared by all genotypes and “dispensable” or variable regions present only in a subset of lines [100,121,122,123]. Classical genotyping approaches based on a single reference are limited by reference bias: variants absent from the reference assembly are either not detected at all or are interpreted incorrectly [124]. The use of pangenomes substantially reduces this bias and enables a more complete description of the true genetic diversity of crop plants [125].

A key technical outcome of the pangenome approach has been graph genomes, in which alternative alleles, structural variants, and insertions are represented as branching paths in a graph rather than as a linear sequence [126]. Read alignment and variant calling in graph space increase sensitivity for detecting SNPs, InDels, and especially structural variation [127,128,129]. For crops with an intensive breeding history (wheat, maize, rice), this enables proper accounting for variants that have been lost in certain reference lines but retained in other cultivars or local populations [130,131,132]. For wheat, an interactive pangenome visualization system based on a graph model was developed, which allows the presence and absence of genes to be clearly displayed across sixteen bread wheat genomes [133]. Such visualization facilitates the comparison of cultivars, helping researchers more quickly identify structural variation, differences in gene content, and regions potentially important for adaptation and breeding.

Pangenome-based genotyping is of particular importance for wild relatives of crops, which often serve as sources of resistance to diseases, stresses, and extreme environmental conditions. When using a single cultivated reference, introgressed segments from wild species may be aligned incorrectly or not detected at all, which complicates their use in breeding [100,131,134]. Pangenome and graph-based approaches enable accurate identification of such regions.

Finally, methods of artificial intelligence and machine learning are playing an increasingly prominent role in data analysis. AI/ML algorithms are used to model complex nonlinear relationships between genotype and phenotype, integrate genomic, transcriptomic, and phenotypic data, and improve the accuracy of genomic predictions [135,136,137]. One of the key directions is phenotype prediction from genotype—models trained on marker data make it possible to predict both quantitative and qualitative plant traits, including yield, stress resistance, flowering time, and others [138,139,140]. In one study, artificial intelligence methods were applied to almond data; Random Forest models achieved a correlation of approximately 0.73 in predicting shelling fraction [141]. Another aspect is comparing the performance of different types of ML models: linear and regularized regression models, ensemble methods (Random Forest, Gradient Boosting), and deep learning models. Lourenço et al. evaluated models on real maize data and showed that in some cases simple regularized methods can compete with more complex ones, especially with a moderate number of markers and limited sample size, as complex models often require large datasets and substantial computational resources [142]. ML is applied in the context of “orphan crops”– plants for which genomic data are scarce—to transfer knowledge from well-studied crops. For example, MacNish et al. [143] showed that machine learning enables the transfer of knowledge from well-studied crops to understudied species, improving trait prediction and accelerating breeding under conditions of limited genomic data.

Despite the growing popularity of machine learning methods in genomic selection, their application is accompanied by a number of methodological limitations. One of the key problems is the so-called “curse of dimensionality,” which is typical for breeding problems where the number of markers (p) greatly exceeds the number of available samples (n) [144,145]. Under such conditions, complex machine learning models can easily overfit and demonstrate high accuracy on training data but limited transferability to independent datasets [146]. Traditional statistical methods, including Bayesian genomic selection models (e.g., BayesA, BayesB, BayesC) and regularized regressions (ridge regression, LASSO), were originally developed to operate under conditions of *p* >> *n* and often show comparable or even more stable predictive performance [145,147].

In addition, many machine learning algorithms belong to the category of “black-box models,” which complicates the biological interpretation of results [148]. In the context of genetic research, it is important not only to predict the phenotype but also to understand which genetic factors underlie the predictions. Therefore, in recent years there has been growing interest in interpretable machine learning methods, such as SHAP (Shapley Additive Explanations), which allow the quantitative assessment of the contribution of individual markers to the model [149]. For example, in the study by Novielli et al., in addition to accurate prediction, SHAP methods were used to identify significant SNPs and genomic regions associated with the trait [141]. Nevertheless, even with the use of such tools, the question remains as to how well the obtained explanations reflect real biological mechanisms.

The speed of implementing genotyping into the breeding cycle and the speed of breeding decision-making depend more on the organization of the end-to-end pipeline [150] than on the choice of a specific genotyping platform per se: how cheaply and at what scale early generations can be genotyped [151], how quickly predictive models can be updated [152], and how genomic signals can be matched with high-frequency phenotypes [153]. An ultra-low-cost genotyping approach obtains extremely sparse coverage per sample—on the order of hundredths of the genome—and then reconstructs dense genotypes via imputation [154]. Its practical value for breeding is that genotyping becomes sufficiently inexpensive to enable continuous, high-throughput screening of thousands to tens of thousands of candidates per cycle. Using intermediate wheatgrass (*Thinopyrum intermedium* (*Host*) *Barkworth & Dewey*) as an example, a pipeline has been presented that is directly oriented toward breeding-program operation: ultra-low coverage skim-seq (approximately 0.01×–0.05×), imputation using STITCH, and subsequent application of these data in genomic selection to predict breeding values in the crop [155].

The emergence of practical haplotype graphs has simplified the reuse of haplotype information from a reference panel within a breeding program, primarily for imputation from low-coverage data. For sorghum, a Practical Haplotype Graph (PHG) has been proposed, demonstrating that even at extremely low coverage of about 0.01×, imputed genotypes suitable for breeding applications can be obtained [156]. In work on creating the Wheat PHG database, it was shown that imputation accuracy can remain high even at very low coverage of about 0.01× (~92% accuracy) [157]. As a result, ultra-low-cost genotyping ceases to be merely an auxiliary tool: genotypes are updated rapidly and at scale, GS models are rebuilt more frequently, and expensive phenotyping can be concentrated on a smaller number of candidates.

Single-cell DNA genotyping in plants remains a niche and methodologically challenging endeavor, whereas single-cell/single-nucleus transcriptomics and other single-cell “omics” are already being applied to dissect complex traits by cell type/trajectory and to build mechanistic links between genetic variation and phenotype [158,159]. An example of the move toward reproducible protocol solutions is a detailed single-cell ATAC-seq protocol for nuclei from maize (*Z. mays*) seedlings, which enables the generation of cell-type-specific chromatin accessibility maps in an agricultural crop [160]. A time-resolved single-cell transcriptomic analysis of Arabidopsis embryo germination has been presented, showing how cell states are formed, and regulatory programs shift as growth is initiated [161]. Single-cell data enable analysis of how genetic variants alter gene regulation and expression in specific cell types and states, helping to explain and predict complex traits that depend on limited tissue and cellular contexts [162,163]. In practical breeding, the near-term role of single-cell approaches is to refine which tissues, stages, and regulatory nodes should be measured and prioritized to improve the transferability of predictions [164].

Integration with phenomics and multi-omics is a direction that makes genomic models more accurate and transferable across environments. For most agronomically important traits, strong dependence on growing conditions and temporal dynamics is typical; therefore, one-time measurements at a single developmental stage often do not reflect the true dynamics of phenotype formation [165]. High-throughput phenomics (HTP) addresses this issue through repeated, standardized observations throughout the growing season [166,167]. Additional gains in informativeness are achieved through multi-omics approaches (transcriptomics, epigenomics/chromatin accessibility, metabolomics), which serve as intermediate layers between genotype and phenotype [168,169]. Such data can act as additional predictors and simultaneously explain why the same genetic background manifests differently under changing environments. Taken together, combining large-scale genotyping with omics approaches forms the basis for predictive breeding of dynamic traits, where models are regularly updated as more data accumulate.

## 6. Genotyping of Complex Genomes and Its Importance in GWAS and Genomic Selection

Genotyping crops with polyploid and/or highly repetitive genomes remains one of the most technically challenging areas of applied plant genomics. For species such as hexaploid wheat (*T. aestivum*), tetraploid cultivated potato (*S. tuberosum*), and allopolyploid rapeseed (*Brassica napus* L.), key constraints are associated with multiple homeologous copies of loci, a high proportion of repeats and paralogs [63,170,171,172]. This complicates both the design of marker panels and the interpretation of NGS data, increasing the risk of erroneous variant calling [173]. Unlike diploids, where genotypes are typically encoded as AA/AB/BB, in tetraploids and hexaploids it is necessary to estimate allele dosage (e.g., for a tetraploid, classes from AAAA to BBBB) [9]. Dosage errors lead to systematic bias in marker-effect estimates, reduced GWAS power, and decreased genomic selection accuracy [174].

The most common strategy for polyploids remains SNP arrays with pre-optimized marker sets that minimize cross-hybridization and ambiguous assignment to homeologs. For example, the Affymetrix Wheat660K array has been used to construct a high-density genetic map in hexaploid wheat, where the large SNP panel provided sufficient genome coverage for high-resolution mapping and subsequent genetic studies [20]. For rapeseed (*B. napus*), similar roles have been played by ~60 K arrays designed to select predominantly single-locus markers in an allotetraploid genome [27]. In particular, the Brassica 60K Infinium SNP array has been described as a universal platform for linkage mapping, association analysis, diversity assessment, and introgression detection, with particular attention to which SNPs are truly “locus-specific” and do not amplify multiple homeologous/paralogous regions simultaneously.

Allele dosage determination in polyploids is usually based on the analysis of allele signal ratios (for example, SNP array intensities or the proportion of reads for the alternative allele in NGS data). Different software packages use different statistical approaches to address this task. For example, polyRAD implements a Bayesian model that estimates posterior genotype probabilities taking into account sequencing depth, sequencing errors, and population structure [175].

The packages updog [176] and fitPoly [177] use models of allele signal distributions to estimate dosage based on the probabilistic distribution of intensities or allele frequencies, which makes it possible to account for genotyping uncertainty. Other tools, such as SuperMASSA [178] or MAPpoly [179], apply probabilistic and EM algorithms for the joint estimation of dosage and segregation parameters in mapping populations.

The transition from rigid discrete genotype calls to probabilistic representations makes it possible to account more appropriately for measurement uncertainty and reduces the likelihood of systematic errors in the analysis of polyploid genomes [180].

Classical single-locus GWAS often underestimates traits with a polygenic architecture and many small-effect loci [181,182]. Multi-locus GWAS (ML-GWAS) models simultaneously account for multiple loci and thereby increase power to detect signals, especially in breeding panels of moderate size [183]. In applied genetics, GWAS results are increasingly interpreted not as single “signals,” but as sets of associated genomic regions, which are then used to refine candidate genes and for functional validation [184]. The key methodological feature of genomic selection (GS) is the use of the entire marker set to predict genomic estimated breeding values (GEBVs), rather than relying on a small number of significant markers. This enables work with traits controlled by many small-effect loci and shifts the emphasis from causal interpretation to predictive accuracy and robustness [185,186]. Therefore, the use of probabilistic genotyping methods and the explicit consideration of genotype uncertainty are regarded as an important step toward improving the reliability of association studies and breeding predictions in polyploid crops.

**Figure 3:**
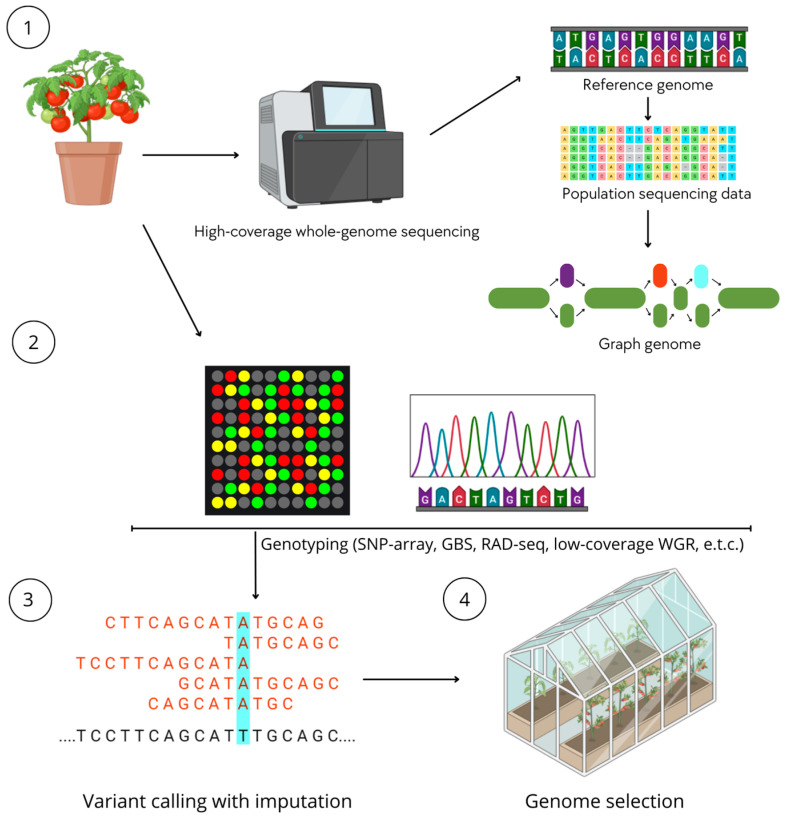
Conceptual diagram of genotyping and its use in genomic selection.

The genotyping methods discussed in this work demonstrate a consistent evolution in plant genomics—from standardized, high-throughput screening of predefined polymorphisms to more flexible and informative strategies for analyzing genomic variation. SNP arrays remain a key tool for high-throughput genotyping due to their high reproducibility, data standardization, and cost efficiency when working with large populations. Their importance is especially high in genomic selection, monitoring the genetic purity of lines, and long-term breeding programs, where data consistency across experiments and generations is critical (Figure 3).

At the same time, reduced-representation and targeted sequencing methods (GBS, RAD-seq, targeted sequencing) have substantially expanded the possibilities for analyzing genetic diversity by enabling work with novel, rare, and population-specific variants that are not accessible with SNP chips. Whole-genome strategies, including WGR, low-coverage WGS, and long-read sequencing, offer the highest resolution for analyzing genetic structure, structural variants, and haplotypes, but are constrained by cost and computational requirements. Their use is particularly justified at the stages of variant discovery, QTL mapping, and reference panel construction.

## 7. What Is Next?

Marker-assisted selection (MAS), which dominated the early stages of molecular plant breeding, focused on using a limited number of loci with large effects. However, these approaches proved to be limited when dealing with quantitative traits controlled by thousands of genetic factors with small effects [185]. Modern plant genotyping is entering a phase in which the key driver of progress is no longer further increases in marker density or refinement of individual platforms, but rather the integration of genomic data into a continuous breeding process. In this context, the concept of genomic estimated breeding value is becoming central as an operational metric. The development of ultra-low-cost approaches enables the analysis of tens of thousands of candidates in each breeding cycle. Under these conditions, GEBV ceases to be a one-time estimate and becomes a dynamic value that is regularly updated as new genotypic and phenotypic data accumulates.

Another important direction of development is the shift from SNP-centric representations of the genome toward haplotype-based and pangenomic models. Pangenomic and graph-based genome representations reduce reference bias and enable proper consideration of structural variants, introgressions from wild relatives, and presence/absence variations, which play a significant role in adaptive traits. In the longer term, this is expected to lead to the development of haplotype-oriented GEBVs that are more robust to transfer across populations and breeding programs.

The limitation of classical GEBV models remains the strong dependence of many agronomically important traits on environmental conditions and plant developmental dynamics [15]. In this context, a key direction for future research is the integration of genomic predictions with high-throughput phenomics and multi-omics data [187,188]. Incorporating phenotypic, transcriptomic, and epigenetic data makes it possible to interpret GEBV as a prediction of a genotype’s response to specific growing conditions. Such models are particularly promising for breeding for abiotic stress tolerance under changing climatic conditions [189,190,191].

Machine learning and artificial intelligence methods are increasingly prominent in GEBV construction, especially for analyzing complex nonlinear relationships and genotype × environment interactions. It has been shown that joint modeling of multiple traits and environments improves predictive accuracy compared with single-trait approaches, particularly for quantitative traits with strong environmental dependence [191,192]. Using maize and wheat as examples, both classical Bayesian multi-trait models and deep learning-based approaches have been shown to effectively exploit correlations among traits and environments to improve estimation of genomic breeding values [193]. At the same time, neural network models achieve comparable accuracy with lower computational costs, whereas statistical models retain an advantage when explicitly modeling genotype × environment interactions. Similar results have been reported in other studies on cereal crops [194], indicating a general trend toward a transition from simple GEBVs to complex multivariate predictive models. However, accumulated experience shows that the advantages of highly complex, poorly interpretable models become evident only when large, well-balanced training datasets are available, which are limited for polyploid, cross-pollinating species [195]. Consequently, there is growing interest in interpretable ML approaches that improve prediction accuracy while identifying gene regions and haplotypes that contribute most to trait variation, thereby bridging predictive breeding and functional genomics.

## 8. Conclusions

Modern approaches to plant genotyping are not competing technologies but form a complementary ecosystem of tools optimized for different stages of the breeding process. SNP arrays provide a reliable foundation for large-scale, standardized analyses; reduced-representation and targeted sequencing methods provide flexibility and access to novel variation, and whole-genome and pangenome strategies provide depth and completeness in representing genetic diversity. A rational combination of these approaches enables effective integration of genomic data into practical breeding.

The future of plant genotyping is not so much about the universal adoption of a single method as about the development of integrated strategies that combine high-quality genotyping data, pangenome models, and artificial intelligence algorithms. This approach opens opportunities for more accurate phenotype prediction, acceleration of breeding cycles, and fuller utilization of the genetic potential of both cultivated plants and their wild relatives, which is of key importance under climate change and increasing demands for agroecosystem resilience.

## Figures and Tables

**Table 1:** Commercial SNP panels for different agricultural crops, with publication activity indicated for each.

Family	Species	Platform	SNP-Array Name	Average Number of Informative Markers	Number of Articles
Rosaceae	Apple (*Malus domestica Borkh*.)	Illumina	RosBREEDApple	$\underline{x}$ = 2476	25
		Axiom	Axiom Apple	$\underline{x}$ = 268,776	14
		Axiom	Axiom Apple (Axiom JKI50kMd)	27,108	1
	Pear (*Pyrus communis* L.)	Axiom	Axiom Pear	NA	1
	Rosa (*Rosa* spp.)	Axiom	Axiom Rose	$\underline{x}$ = 20,048	19
	Peach (*Prunus persica* (L.) *Batsch*)	Illumina	RosBREEDPeach	$\underline{x}$ = 3246	4
	Cherry (*Prunus* spp.)	Illumina	RosBREEDCherry	$\underline{x}$ = 1855	19
	Strawberry (*Fragaria* spp.)	Axiom	Axiom Strawberry 50 K (Axiom Fana_SNP)	$\underline{x}$ = 24,145	3
		Axiom	Axiom Strawberry (i35)	$\underline{x}$ = 11,769	22
		Axiom	Axiom Strawberry (IStraw90K)	$\underline{x}$ = 10,416	21
Poaceae	Maize (*Zea mays* L.)	Illumina	MaizeSNP50	$\underline{x}$ = 28,652	120
		Axiom	Axiom Maize6H (60 K)	$\underline{x}$ = 30,979	7
		Axiom	Axiom Maize	$\underline{x}$ = 429,498	19
	Wheat (*T. aestivum*)	Axiom	Axiom TaNG1.1	12,490	1
		Axiom	Axiom Wheat HD (Bristol)	546,299	2
		Illumina	90 K iSelect	$\underline{x}$ = 16,484	137
	Barley (*Hordeum vulgare* L.), Wheat (*T. aestivum*)	Illumina	Wheat-Barley40K	$\underline{x}$ = 16,617	9
	Rice (*Oryza sativa* L.)	Illumina	RiceLD	$\underline{x}$ = 981	3
		Illumina	RiceSNP50	$\underline{x}$ = 35,597	8
		Axiom	Axiom Rice	NA	0
	Rye (*Secale cereale* L.)	Axiom	Axiom Rye	NA	1
	Barley (*H. vulgare*)	Illumina	Barley50K Consortium Array (barley 50 K iSelect)	$\underline{x}$ = 26,869	41
Solanaceae	Tomato (*Solanum lycopersicum* L.)	Illumina	SolCAP Tomato 2013	$\underline{x}$ = 5967	26
		Axiom	Axiom Tomato	$\underline{x}$ = 29,457	5
	Pepper (*Capsicum annuum* L.)	Illumina	TraitGenetics Pepper Consort.	NA	0
	Potato (*Solanum tuberosum* L.)	Illumina Illumina	GGP Potato-24	$\underline{x}$ = 16,737	4
			SolCAP 8303	$\underline{x}$ = 4333	28
Fabaceae	Soybean (*Glycine max* (L.) Merr.)	Illumina	BARCSoySNP6k	$\underline{x}$ = 3003	56
		Axiom	Axiom Soybean	$\underline{x}$ = 51,758	47
	Pea (*Pisum sativum* L.)	Illumina	GenoPea INRA 13.2 K	$\underline{x}$ = 9480	7
	Lentil (*Lens culinaris Medik.*), Pea (*P. sativum*), Chickpea (*Cicer arietinum* L.), Lupin (*Lupinus* L.)	Illumina	Pulses Array	$\underline{x}$ = 13,216	3
	Peanut (*Arachis hypogaea* L.)	Axiom	Axiom Peanut (Arachis1, Arachis2)	$\underline{x}$ = 9089	39
Malvaceae	Cotton (*Gossypium* spp.)	Illumina	CottonSNP63K	$\underline{x}$ = 13,273	33
		Illumina	CottonSNP80K	$\underline{x}$ = 43,901	21
		Axiom	Axiom Cotton	NA	0

NA—not available. $\underline{x}$—mean value.

**Table 2:** Custom SNP panel designs for main agricultural crops.

Species/Number of Markers	1–12 K	Average Number of Informative Markers	13 K–45 K	Average Number of Informative Markers	46 K–95 K	Average Number of Informative Markers	96 K+	Average Number of Informative Markers
Maize (*Z. mays*)	31	$\underline{x}$ = 978	3	$\underline{x}$ = 19,000	3	1653	9	$\underline{x}$ = 184,800
Rice (*O. sativa*)	19	$\underline{x}$ = 2051	3	NA	1	NA	0	NA
Wheat (*T. aestivum*)	2	NA	4	$\underline{x}$ = 16,888	1	NA	2	92,166
Apple (*M. domestica*)	2	$\underline{x}$ = 2832	2	$\underline{x}$ = 13,793	0	NA	0	NA
Cotton (*Gossypium* spp.)	0	NA	2	$\underline{x}$ = 21,898	0	NA	0	NA
Pepper (*C. annuum*)	1	27	3	$\underline{x}$ = 7313	0	NA	0	NA
Pear (*P. communis*)	4	$\underline{x}$ = 807	0	NA	1	66,616	2	166,335
Peach (*P. persica*)	3	$\underline{x}$ = 3015	0	NA	0	NA	0	NA
Barley (*H. vulgare*)	5	$\underline{x}$ = 1868	0	NA	0	NA	0	NA
Tomato (*S. lycopersicum*)	6	$\underline{x}$ = 2836	1	NA	0	NA	0	NA
Soybean (*G. max*)	4	$\underline{x}$ = 730	0	NA	1	47,337	1	128
Pea (*P. sativum*)	3	$\underline{x}$ = 606	0	NA	0	NA	0	NA

NA—not available. $\underline{x}$—mean value.

## Data Availability

No new data were created or analyzed in this study. Data sharing is not applicable to this article.

## Supplementary Materials

The following supporting information can be downloaded at: https://www.mdpi.com/article/10.3390/life16030521/s1. Table S1—Table listing publications using selected commercial SNP panels. Table S2—Table listing publications using selected custom SNP panels.
